# Molecular detection of *Leishmania* spp. in dogs and a cat from Doha, Qatar

**DOI:** 10.1186/s13071-019-3394-y

**Published:** 2019-03-26

**Authors:** Clara Lima, Vito Colella, Maria Stefania Latrofa, Luís Cardoso, Domenico Otranto, Ana Margarida Alho

**Affiliations:** 1 Parkview Pet Center and Cityview Veterinary Clinic, Doha, Qatar; 2 Dipartimento di Medicina Veterinaria, University of Bari, Valenzano, Italy; 3 Department of Veterinary Sciences and Animal and Veterinary Research Centre (CECAV), University of Trás-os-Montes e Alto Douro (UTAD), Vila Real, Portugal; 4 CIISA, Faculty of Veterinary Medicine, University of Lisbon (ULisboa), Lisbon, Portugal

**Keywords:** Cat, Dog, *Leishmania*, Qatar, Vector-borne diseases, Zoonosis

## Abstract

**Background:**

Canine and feline leishmanioses are increasingly reported worldwide and represent a threat to both animal and human health. Despite their relevance, data about leishmanioses in companion animals in the east-central part of the Arabian Peninsula are unavailable. Therefore, we investigated the occurrence of *Leishmania* spp. in dogs and cats from Qatar.

**Methods:**

From March 2016 to May 2018, 199 pets (120 dogs and 79 cats) living in Doha or its outskirts were included in this study. From each animal a blood sample was collected and tested for *Leishmania* spp. by quantitative real-time PCR.

**Results:**

Out of the 199 animals, four (2.0%) were positive for *Leishmania* spp., including three dogs (2.5%) and one cat (1.3%).

**Conclusions:**

All positive animals were born in Qatar and had not travelled overseas, suggesting that infection was locally acquired. Considering the occurrence of *Leishmania* spp. and its potential impact on the health of animals and humans, it is crucial to increase scientific knowledge in order to plan screening and regular prophylaxis against sand fly vectors to reduce the risk of infection.

## Background

Leishmaniosis is recognized by the World Health Organization (WHO) as a neglected tropical disease that represents a major public health threat worldwide [1, 2]. The disease is endemic in a variety of biogeographical environments with clinical presentations varying according to the causative species of *Leishmania.* As stated by the WHO, human leishmanioses have been reported in the Arabian and Gulf Peninsulas, namely Iraq, Jordan, Oman, Saudi Arabia, Syria and Yemen. Nevertheless, there are no case reports of autochthonous leishmaniosis in Qatar or in the neighboring United Arab Emirates and Bahrain [3, 4].

Qatar is a country located in the Persian Gulf, which extends northward from the main Arabian Peninsula and borders Saudi Arabia. As a result of substantial immigration, Qatar has experienced a fast demographic growth and an associated increase in the number of pets [5], which may have enhanced the risk of exotic vector-borne diseases (VBD).

In a previous study, canine and feline VBD were diagnosed in autochthonous populations of domestic dogs and cats in Qatar [6]. As no data are currently available regarding leishmaniosis in Qatar, we conducted a study to molecularly assess the occurrence of canine and feline infections with *Leishmania* spp. in the country.

## Methods

### Study location

Qatar is a peninsula in the Persian Gulf, 180 km long and 55–85 km wide, covering an area of approximately 11,000 km^2^, with most of its territory made of sand desert. It has an arid climate where rainfall is negligible and confined to the winter months, mainly in the form of sporadic heavy storms and downpours. Summers are characterized by high temperatures (average maximum air temperature in the hottest months, i.e. from May to October, around 40 °C), strong winds and high relative humidity. The population was estimated in 2017 to be 2,743,932 inhabitants, with the capital city Doha harboring a large percentage of the total population [5].

### Animal sampling

From March 2016 to May 2018, 199 owned and stray-rescued pets (94 and 26 dogs, respectively, and 66 and 13 cats, respectively) living in Doha or its outskirts were randomly sampled. Dogs and cats were presented at Parkview Pet Center and Cityview Veterinary Clinic (Doha, Qatar) for health checks or rabies antibody titration prior to pet relocation or adoption.

A complete record was kept for each sampled animal, including species, age, sex, breed, place of birth (whenever available), lifestyle and the reasons for admissions at the veterinary clinic. Signalment of the sampled pets is summarized in Table 1.

**Table 1:** Signalment of sampled pets from Doha, Qatar

	Dogs	Cats
Males (*n*)	72	44
Females (*n*)	48	35
Age range (months)	2–156	4–216
Median age (months)	36	24
Interquartile range (months)	24–72	12–60
Breed (*n*)	Mixed-breed (45), Saluki (30), German Shepherd (12), Siberian Husky (7), Maltese (5), Rottweiler (2), Pug (2), Yorkshire Terrier (2), Borzoi (2), Golden Retriever (2), Belgian Shepherd (1), Cocker Spaniel (1), Collie (1), Dobermann (1), Labrador Retriever (1), Pekingese (1), Poodle (1), Rhodesian Ridgeback (1), Spitz (1), St. Bernard (1), West Highland White Terrier (1)	Domestic short-haired (62), domestic long-haired (7), Persian (6), Bengal (2), Himalayan (1), Maine Coon (1)
Total (*n*)	120	79

From each animal whole blood samples were collected by venipuncture of the cephalic and/or jugular veins, in EDTA tubes or spotted onto filter papers and stored at -20 °C until use.

### Molecular procedures

Genomic DNA was extracted using the QIAamp DNA Micro Kit or DNeasy Blood & Tissue Kit (Qiagen, Hilden, Germany) for blood stored on dried spots or in EDTA, respectively, according to the manufacturer’s instructions. DNA of *Leishmania* spp. was detected by quantitative real-time PCR (qPCR) as described elsewhere [7]. All samples were tested in duplicate and positive (DNA of pathogen-positive blood sample) and negative [pathogen-negative blood sample and no-template control (NTC)] reference samples were included in each run.

## Results

Out of the 199 pets tested, four (2.0%) scored positive for *Leishmania* spp., including three dogs (2.5%) and one cat (1.3%). Among the positive dogs, two were Salukis and one was mixed-breed, with ages ranging from 24 to 36 months. All of them had outdoor activity, as they had been regularly taken for walks in the city and also in the mangroves and deserts, on the outskirts of Doha. Of the three animals, one was admitted because of chronic diarrhea and was diagnosed with exocrine pancreatic insufficiency, the second was an errant dog rescued from the street, and the last was admitted for an elective neutering surgery and had an unremarkable physical condition (Table 2). The only *Leishmania* spp*.-*positive cat was a domestic short-haired, 10-month-old. The cat had been a stray animal that was rescued from the streets and brought to the hospital for a health check by the owners (Table 2).

**Table 2:** Data regarding *Leishmania* spp.-positive dogs (2.5%) and cats (1.3%) from Doha, Qatar

Species	Age (months)	Sex	Breed	Place of birth	Lifestyle	Reason for veterinary appointment	Ct value^a^
Dog	24	M	Saluki	Qatar	Indoor/outdoor	Chronic diarrhea due to exocrine pancreatic insufficiency	36.30
Dog	36	M	Mixed	Qatar	Indoor/outdoor	Rescued from the street, history of car crash and hind limb paralysis, chronic kidney disease	35.55
Dog	nd	F	Saluki	Qatar	Indoor/outdoor	Elective surgery (neutering)	36.34
Cat	10	F	DSH	Qatar	Outdoor	Rescued from the street, bilateral blindness	35.88

^a^Cycle threshold values at the real-time PCR

*Abbreviations*: DSH, domestic short-haired; F, female; M, male; nd, not determined

## Discussion

To the best of our knowledge, this is the first detection of *Leishmania* spp. infection in dogs and cats from Qatar.

Dogs are the primary reservoirs of *Leishmania infantum*, the main causative agent of canine leishmaniosis (CanL), representing the major source of infection for human zoonotic visceral leishmaniosis, through phlebotomine sand fly bites. More recently, cats have also been diagnosed with leishmaniosis and identified as potential secondary reservoirs of *L. infantum* [8, 9]. Meanwhile, *Leishmania major* and *L. tropica* have been pointed out as the most common agents of cutaneous leishmaniosis in the Middle East in humans, while they are considered rare causes of CanL in the Old World. However, cutaneous manifestations of *L. major* in a dog and *L. major* and *L. tropica* in two other dogs from Israel have been reported [10, 11].

A previous survey [12] identified a gap of understanding and lack of awareness of the pet owner population in Qatar about the concept of zoonotic diseases and prophylactic measures to minimize their risks. Due to the zoonotic potential of leishmaniosis and the role of canine hosts as reservoirs, emphasis must be put into education of local pet owners on how to prevent leishmaniosis in their pets, an endeavor that requires collaborative efforts from human and animal health systems. Canine leishmaniosis may be a serious threat for animals travelling to and from countries where awareness on the disease and related preventative measures are unavailable [13–15]. Hence, further epidemiological studies, including a larger population of animals from Qatar, should be performed to obtain data on the distribution of VBD-causing pathogens in the country, and to plan prevention programs wherever necessary. Indeed, establishing control strategies against *Leishmania* spp. infections in humans and animals requires a thorough assessment of the prevalence of infection and investigation on the occurrence of species of phlebotomine sand flies with known competency as vectors.

## Conclusions

The finding that all the positive animals were born in Qatar and had not travelled outside the country suggests that infection was locally acquired. This is, therefore, to the best of the authors’ knowledge, the first report of infection with *Leishmania* spp. in pets from this country. Considering the impact of leishmaniosis on the health of companion animals, it is vital to increase scientific knowledge about its epidemiological situation, and ensure routine screening and regular prophylaxis, in order to decrease exposure and contact with these pathogens.

## Abbreviations

CanL: canine leishmaniosis
VBD: vector-borne diseases
WHO: World Health Organization
